# Surgical Outcomes and Complications of Distal Nasal Reconstruction: A Systematic Review and Meta-Analysis

**DOI:** 10.3390/jcm14227983

**Published:** 2025-11-11

**Authors:** Giovanni Salzano, Veronica Scocca, Antonio Romano, Luigi Angelo Vaira, Jerome R. Lechien, Fabio Maglitto, Marzia Petrocelli, Giovanni Dell’Aversana Orabona

**Affiliations:** 1 Maxillofacial Surgery Unit, Department of Neurosciences, Reproductive and Odontostomatological Sciences, University Federico II of Naples, Via Pansini 5, 80131 Naples, Italy; giovannisalzanomd@gmail.com (G.S.);; 2 Maxillofacial Surgery Operative Unit, Department of Medical, Surgical and Experimental Sciences, University of Sassari, 07100 Sassari, Italy; 3 Department of Surgery, University of Mons, 7301 Mons, Belgium; 4 Oral and Maxillo-Facial Unit, AUSL Bologna Bellaria-Maggiore Hospital, 40139 Bologna, Italy

**Keywords:** nasal reconstruction, local flaps, free flaps, skin grafts, functional outcomes, aesthetic outcomes, surgical morbidity

## Abstract

**Background:** Reconstruction of the distal nose represents a major surgical challenge due to the aesthetic and functional relevance of this subunit. Various techniques—including local, regional and free flaps and grafts—have been described, but high-quality evidence comparing outcomes remains limited. **Methods:** Following PRISMA guidelines, a systematic review was conducted using PubMed/MEDLINE, Cochrane Library, Scopus, Embase, and Google Scholar. A single-arm meta-analysis was performed to evaluate complications, revision surgeries, and aesthetic and functional outcomes. Secondary outcomes included flap necrosis, revision procedures, and airway function. **Results:** Forty articles were included in the qualitative synthesis and 38 in the quantitative analysis, from an initial 587. The analysis involved 1362 patients (mean age 60.6 years) undergoing distal nasal reconstruction, most commonly for malignancy. The pooled complication rate was 11%, and was highest with regional flaps (26%). Flap/graft necrosis occurred in 5% of free flaps and 2% of regional flaps, with none reported for local flaps or grafts. Revision surgery was required in 7% overall, but was greater with mixed flaps (11%), compared to graft (3%), local (1%), and regional (0%) techniques. Dermabrasion and secondary contouring were infrequent. Aesthetic outcomes were inconsistently reported, precluding meta-analysis. **Conclusions:** This systematic review and meta-analysis provide an overview of reconstructive options and outcomes for distal nasal defects. Local and regional flaps remain the most reliable and versatile solutions for small-to-moderate, partial-thickness defects, offering low complication and revision rates. Free flaps, while essential for extensive or full-thickness reconstructions, are associated with greater morbidity and revision burden. Standardised reporting of outcomes relative to defect size and thickness is required to guide evidence-based decisions.

## 1. Introduction

The nose, centrally positioned on the face, is a defining feature of facial aesthetics, self-expression and individual identity. Its complex three-dimensional structure makes nasal reconstruction one of the most challenging tasks in facial plastic surgery, particularly in the distal regions—including the tip, soft triangle, alar rim and lateral alar area—where both airway patency and aesthetic symmetry must be preserved. Successful reconstruction requires restoring the nasal shape, achieving colour and texture harmony with the surrounding skin, and adhering to the principle of nasal subunits [1,2,3].

A wide range of reconstructive options is available, depending on the defect size, depth, location and subunit involvement. Small, superficial defects may be amenable to primary closure or skin grafting, although grafts often lead to a suboptimal colour and texture match [4]. Local flaps—such as rotation, advancement and transposition flaps, including specialised designs like bilobed, trilobed, spiral and quadrilobed flaps—are generally preferred for larger or more complex defects due to the superior tissue match in terms of thickness, sebaceous quality and contouring [5].

When local tissue is insufficient, regional flaps—including nasolabial, frontonasal and paramedian forehead flaps—offer a reliable vascularity and coverage, particularly for extensive or full-thickness defects [5,6]. Free flaps or composite grafts (skin, cartilage or skin–fat–fascia) are reserved for rare cases where local and regional options are inadequate. This reconstructive ladder allows surgeons to tailor their approach to each defect while optimising both functional and aesthetic outcomes [7].

The aim of this systematic review and meta-analysis is to provide an overview of the surgical, functional, and aesthetic outcomes of distal nasal reconstruction using different flap techniques, highlighting the range of approaches rather than determining a single optimal method.

## 2. Materials and Methods

This study followed the Preferred Reporting Items for Systematic Reviews and Meta-Analyses (PRISMA) guidelines. Since it involved a review of previously published studies, neither ethics approval nor informed consent was required. Additionally, the review was registered in the PROSPERO database under the ID number CRD420251133928. The completed PRISMA checklist [8] is provided as Supplementary Materials (File S1).

### 2.1. Eligibility Criteria

This systematic review and meta-analysis was carried out in accordance with PICOTS: Patients (P), patients who had undergone distal nose reconstruction (ala, tip, columella, lower dorsum and sidewall); Intervention (I) local flap, loco-regional flap, free flap; skin graft reconstruction; Comparison (C), between type of flap used for the reconstruction; Outcomes (O) surgical, functional and aesthetic outcomes; Timing (T), no restrictions were applied; Study design (S), retrospective and prospective cohort studies, case–control and cross-sectional studies and randomised controlled trials (RCTs).

Studies were excluded if: they were not available in full-text form; included fewer than 4 patients; were presented as either a review, case report, conference abstract, letter to the editor or book chapter; addressed total nasal reconstruction or reconstruction of the upper part of the dorsum. No restrictions on the publication date were applied, but articles had to be published in a peer-reviewed journal.

### 2.2. Search Strategy

The study search included the PubMed/MEDLINE, Cochrane Library, Scopus, Embase and Google Scholar databases. The search was conducted independently by two investigators (V.S. and G.S.). Relevant keywords, phrases, and MeSH terms were tailored to meet the specific requirements of each individual database. (Table S1) The search strategy used was “(distal nose OR distal third nose) AND (reconstruction OR reconstructive surgery)”. Next, a cross-reference search of the selected articles was conducted using the snowballing method to ensure the retrieval of all possible studies. The electronic database search was conducted from 2 August 2025 to 15 August 2025.

### 2.3. Data Collection Process

References from the identified databases were merged, and duplicates were removed using the reference management software EndNote^®^ 21 (version 21.5). The articles were screened for relevance based on title and abstract, with those deemed appropriate selected for a full-text review. Any discrepancies between the screening authors were resolved through discussion until a consensus was achieved. Systematic data extraction from the studies included was undertaken using a structured form, with data archived in a customised Excel^®^ (Microsoft Corp, Seattle, WA, USA) spreadsheet. One author (V.S.) independently compiled a standardised form to extract the following characteristics from the studies included: authors, year of publication, country, study design, surgical department, surgeon information, number of patients, mean age, aetiology, type of cutaneous reconstruction, defect location and depth, mean defect size, number of operative stages, mean follow-up time, tumour recurrence and reported outcomes. The accuracy of the extracted data was verified by a second author (G.S.).

### 2.4. Data Synthesis and Analysis

All articles included in the qualitative analysis were then included in the meta-analysis.

The mean defect size was reported in centimetres for all the studies included. Continuous variables were reported as mean values whenever available; if the mean was not provided, the median was extracted and reported.

Aesthetic outcomes were assessed qualitatively rather than quantitatively, due to the substantial heterogeneity in the methods used for aesthetic evaluation across the studies.

For each study, the number of patients experiencing complications was extracted. Specific complications included flap/graft necrosis, wound infection or dehiscence, alar notching, contour irregularities or contraction, nasal valve collapse or obstruction, pincushion or trapdoor deformities, and other reported events. Flap or graft necrosis specifically refers to partial or total tissue loss of the reconstructed flap or graft.

A single-arm meta-analysis was performed in relation to any complications, both overall and for each specific type when feasible, as well as for revision surgeries and dermabrasion, stratified according to the reconstructive technique (‘local’, ‘graft’, ‘free’, ‘mixed’, ‘regional’). The term ‘mixed’ indicated studies in which more than one reconstructive approach was used (free flap + local flap/regional flap/graft). The results were presented as pooled estimates with 95% CIs, and a forest plot was generated for each outcome. To stabilise variance in the analysis of proportions, the Freeman–Tukey double arcsine transformation was performed.

Cochran’s Q test was applied to assess heterogeneity between the studies, and I^2^ was calculated as a measure of heterogeneity. The I^2^ value represents the percentage of total variation between the studies caused by heterogeneity rather than by chance. According to the Cochrane criteria, values from 0% to 40% may signify a low heterogeneity, 30% to 60% a moderate heterogeneity, 50% to 90% a substantial heterogeneity, and 75% to 100% a considerable heterogeneity.

A random-effects model was used for all the meta-analyses, on the assumption that the true effect size may vary between studies due to differences in the study populations, methodologies or other sources of variability. This model accounts for both within-study and between-study heterogeneity, providing more conservative and generalizable effect estimates.

All the analyses were performed using the R software for statistical computing (R version 4.4.2; “meta” and “dmetar” packages). Statistical significance was defined as *p* < 0.05.

### 2.5. Risk of Bias Assessment

Two authors (V.S. and G.S.) assessed the quality of each study using the Newcastle–Ottawa Quality Assessment Scale, since all the studies included were performed according to observational cohort or case–control designs. To evaluate any potential publication bias, a funnel plot was generated based on the effect size of each outcome, and the degree of symmetry was formally assessed using Egger’s linear regression test. Sensitivity analyses were performed for each outcome to assess the robustness of pooled estimates and explore potential sources of heterogeneity.

## 3. Results

### 3.1. Study Selection

**Figure 1:**
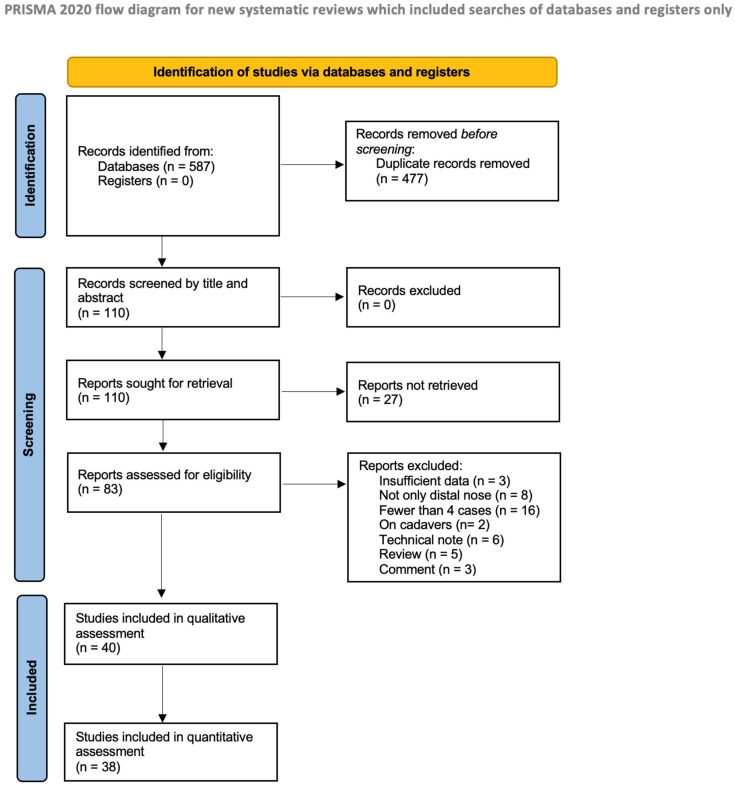
PRISMA flow diagram.

The study selection process is summarised in Figure 1. Following a comprehensive search and removal of duplicates, 587 articles were identified. After title screening, 110 articles remained, of which 83 were assessed in full text. Three studies were excluded due to insufficient data [9,10,11]; 16 because of the small sample size (fewer than 4 patients) [12,13,14,15,16,17,18,19,20,21,22,23,24,25,26,27]; six as technical notes [28,29,30,31,32,33]; five as review articles [34,35,36,37,38]; and three as comments [39,40,41]. Eight studies were excluded because they did not exclusively address distal nasal defect reconstruction [42,43,44,45,46,47,48,49], and two studies because they were conducted on cadaver models [50,51]. Finally, 40 publications were included in the qualitative synthesis, and 38 in the quantitative synthesis (meta-analysis), as two studies reported the use of different flaps but did not stratify outcomes according to flap type [52,53].

### 3.2. Study Characteristics

The general characteristics of the studies are shown in Table 1 [5,52,53,54,55,56,57,58,59,60,61,62,63,64,65,66,67,68,69,70,71,72,73,74,75,76,77,78,79,80,81,82,83,84,85,86,87,88,89,90]. All the studies included, when reported, were retrospective. Four studies were published in the 1990s [54,55,56,57], eight in the 2000s [79,80,81,82,83,84,85,86], twenty-one in the 2010s [5,52,53,61,62,63,64,65,66,67,68,69,70,71,72,73,74,75,76,77,78], and seven in the 2020s [54,55,56,57,58,59,60].

### 3.3. Study Results

A total number of 1.362 patients (males: 484/963, 50.2%) with a mean age of 60.6 years (*n* = 672/1362, 95%CI 18–78) were included. Most of the patients had presented a malignant condition, especially basal cell carcinoma (*n* = 592/725, 81.6%) and squamous cell carcinoma (*n* = 92/725, 12.7%).

Local flaps were described in 17 studies, including bilobed, trilobed, quadrilobed, spiral, dorsal nasal, nasal sidewall and nasal tip flaps. Regional flaps were reported in 14 studies, comprising forehead, nasolabial and frontonasal flaps. Free flaps were documented in only two studies, and consisted of ear free flaps. Cartilage and skin grafts were used in five studies. Finally, two studies reported the use of regional and free flaps within the same cohort.

Only seven studies reported the rate of tumour recurrence [6,69,70,75,77,78], and among these, only Ghassemi et al. [77] documented recurrence, with a rate of 5 out of 21 cases (23.8%).

### 3.4. Complication Rate

**Figure 2:**
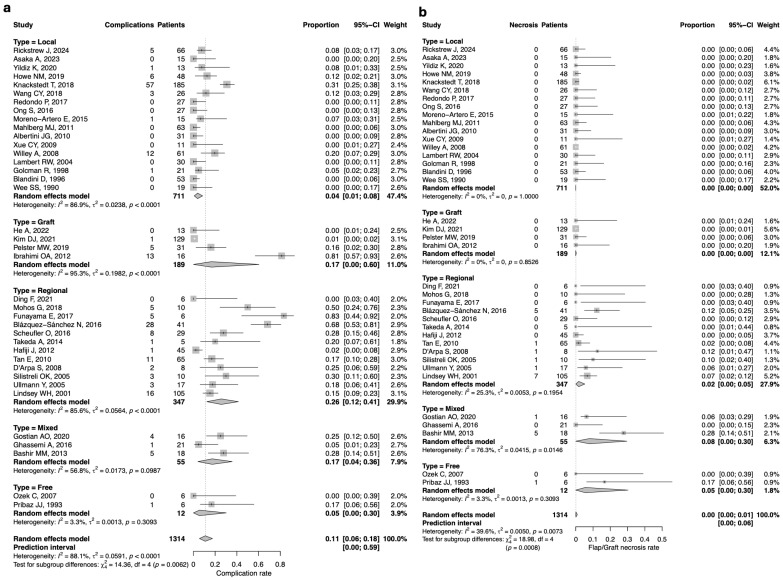
(**a**) Forest plot for complication rate according to the reconstructive technique; (**b**) Forest plot for flap/graft necrosis rate according to the reconstructive technique [5,52,53,54,55,56,57,58,59,60,61,62,63,64,65,66,67,68,69,70,71,72,73,74,75,76,77,78,79,80,81,82,83,84,85,86,87,88,89,90].

An overall complication rate of 11% (*n* = 144/1314; 95%CI: 6–18) was observed, with a substantial between-study heterogeneity (I^2^ = 88.1%, Q = 0.0591, *p* < 0.0001). When stratified by reconstructive technique, the highest complication rate was found in the regional flap group (26%; *n* = 90/347; 95%CI: 12–41; I^2^ = 85.6%, Q = 0.0564, *p* < 0.0001). In contrast, the graft and mixed groups all showed a complication rate of 17% (19/189; 95% CI: 0–60; 10/55; 95% CI: 4–36; and 39/248; 95% CI: 6–18, respectively), again with a substantial heterogeneity. The local flap group showed a complication rate of approximately 4%, while the free flap group reported a slightly higher rate of 5%. However, it should be noted that the free flap subgroup included only 12 patients overall, limiting the strength of this estimate and the possibility of drawing definitive conclusions. (Figure 2a)

In relation to specific complications, such as alar notching, nasal valve collapse/obstruction and pincushion/trapdoor deformity, such events were rarely reported, with most studies documenting no cases. (Table 2) Consequently, a pooled meta-analysis could not be performed.

Regarding flap/graft necrosis, no cases were documented in the local and graft groups. The mixed group had an 8% necrosis rate (*n* = 6/55; 95%CI: 8–30), with a substantial between-study heterogeneity (I^2^ = 76.3%, Q = 0.0415, *p* = 0.146). The free flap group showed a 5% necrosis rate (*n* = 1/12; 95%CI: 0–10), with a low heterogeneity (I^2^ = 3.3%, Q = 0.0013, *p* = 0.3093). Conversely, the regional flap group had a 2% necrosis rate (*n* = 16/347; 95%CI: 0–5), also with low heterogeneity (I^2^ = 25.3%, Q = 0.0053, *p* = 0.1954). (Figure 2b).

**Figure 3:**
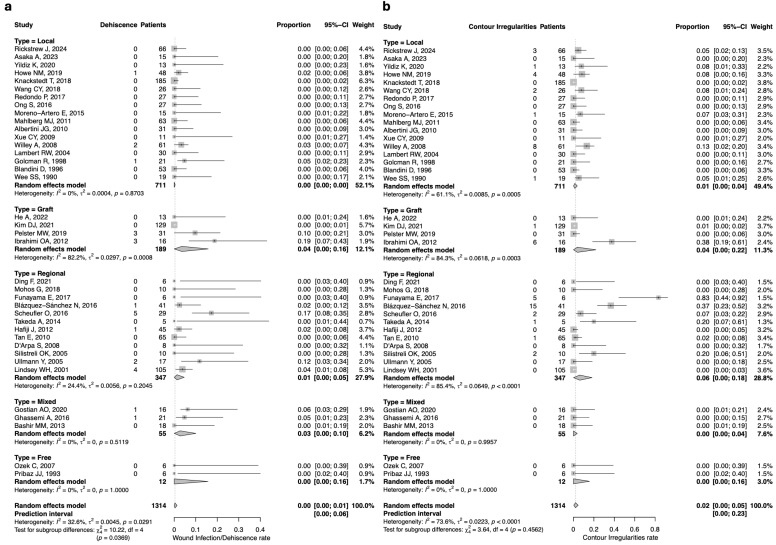
(**a**) Forest plot for wound infection/dehiscence rate according to the reconstructive technique; (**b**) Forest plot for contour irregularities rate according to the reconstructive technique [5,52,53,54,55,56,57,58,59,60,61,62,63,64,65,66,67,68,69,70,71,72,73,74,75,76,77,78,79,80,81,82,83,84,85,86,87,88,89,90].

The pooled estimate of wound infection/dehiscence was 0% in both the local and free flap groups. Similar rates were observed in the remaining groups: the mixed flap and graft groups reported a 3% and 4% rate, respectively (*n* = 2/55; 95%CI: 0–10 and *n* = 6/189; 95%CI: 0–16, respectively), with low between-study heterogeneity for the regional flap group. The regional group also showed low heterogeneity, with a 1% rate of wound infection/dehiscence (*n* = 13/347; 95%CI: 0–5). (Figure 3a).

The pooled estimate of contour irregularities/contraction was 0% in both the mixed and free flap groups, with no between-study heterogeneity. The local group showed a rate of 1% (*n* = 20/711; 95%CI: 0–4), with moderate between-study heterogeneity (I^2^ = 61.1%, Q = 0.0085, *p* = 0.0005). The highest rate was observed in the regional flap group, estimated at 11% (*n* = 26/347; 95%CI: 0–18), with substantial heterogeneity (I^2^ = 85.4%, Q = 0.0649, *p* < 0.0001). The graft group reported a slightly lower rate of 4% (*n* = 7/189; 95%CI: 0–22), also with substantial heterogeneity. (Figure 3b).

### 3.5. Revision Surgery

**Figure 4:**
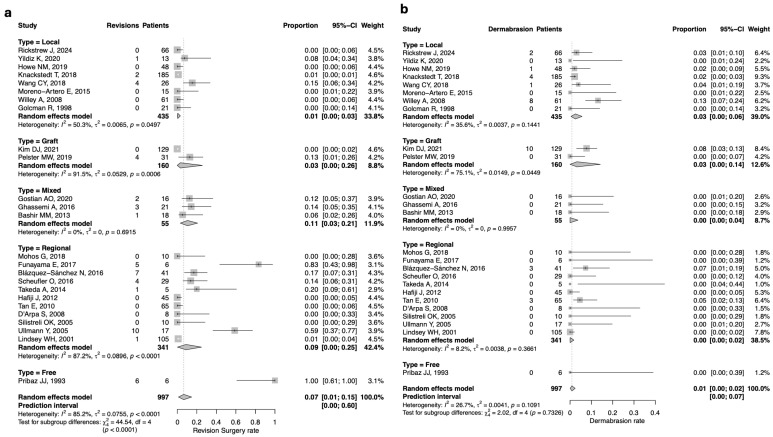
(**a**) Forest plot for revision surgeries rate according to the reconstructive technique; (**b**) Forest plot for dermabrasion rate according to the reconstructive technique [5,52,53,54,55,56,57,58,59,60,61,62,63,64,65,66,67,68,69,70,71,72,73,74,75,76,77,78,79,80,81,82,83,84,85,86,87,88,89,90].

The overall pooled estimate of revision surgery was 7% (*n* = 70/997; 95%CI: 1–15), with substantial between-study heterogeneity (I^2^ = 85.2%, Q = 0.0755, *p* < 0.0001). The highest rate was observed in the mixed flap group, with a revision rate of 11% (*n* = 6/55; 95%CI: 3–21) and no heterogeneity. Similarly, the regional flap group showed a pooled estimate of 9% (*n* = 28/341; 95%CI: 0–25), with substantial heterogeneity (I^2^ = 87.2%, Q = 0.0896, *p* < 0.0001). Lower rates were found in the graft group (3%; *n* = 4/160; 95%CI: 0–26), which exhibited high heterogeneity (I^2^ = 91.5%, Q = 0.0529, *p* = 0.0006), and in the local group (1%; n = 7/435; 95%CI: 0–3), with moderate heterogeneity (I^2^ = 50.3%, Q = 0.0065, *p* = 0.0497). Since only one study [59] in the free flap group reported revision, no pooled esteem was calculated; however, the revision surgery rate in that study was 100% (*n* = 6/6; 95%CI: 61–100). (Figure 4a).

The pooled estimate of dermabrasion was 0% was reported in both the regional and mixed flap groups, with no between study heterogeneity for the mixed flap group. A rate of 3% was reported in the local flap and graft groups, with a moderate between study heterogeneity in the local group (I^2^ = 35.6%, Q = 0.003, *p* = 0.1441). Since only one study in the free flap group [59] reported data, no pooled estimate was calculated; no cases of dermabrasion were documented in that study. (Figure 4b).

In relation to other revision treatments, such as intralesional corticosteroid injection and laser therapy, these were rarely reported, with most studies documenting no cases (Table 3). Therefore, a pooled meta-analysis could not be performed.

### 3.6. Aesthetic Outcomes

Table 4 provides a qualitative assessment of all 40 studies included. A pooled quantitative analysis of aesthetic outcomes was not feasible due to the heterogeneity of reporting methods and scales across studies. Additionally, several studies either did not report aesthetic outcomes or did not provide data that could be quantified [53,54,55,56,58,63,64,66,67,73,80,82,87,90].

### 3.7. Risk of Bias Assessment

The methodological quality of the included studies was assessed using the Newcastle-Ottawa Scale (NOS), with scores ranging from 5 to 9 and a mean score of 7.2 (Table S3). Common sources of bias were primarily related to selection of cohorts and comparability of study groups, while outcome assessment was generally well-reported. These findings suggest that most studies were of moderate to high quality; however, caution is warranted when interpreting the pooled results, as potential biases in study design and reporting could influence the overall estimates.

Leave-one-out sensitivity analysis in Figure S1 confirmed that the pooled estimates for wound infection/dehiscence, flap/graft necrosis, and revision surgery remained stable, with no single study exerting a disproportionate influence on the overall results. This indicates that the findings were robust and not driven by individual studies. By contrast, the pooled estimate for dermabrasion was more sensitive to the exclusion of individual studies [62,82], suggesting that this outcome was largely influenced by a few reports and should therefore be interpreted with caution.

**Figure S1** -**a.** Leave-one-out sensitivity analysis for pooled complication rate; **-b.** Leave-one-out sensitivity analysis for pooled flap/graft necrosis rate; -**c.** Leave-one-out sensitivity analysis for pooled wound infection/dehiscence rate; -**d.** Leave-one-out sensitivity analysis for pooled contour irregularities rate; -**e.** Leave-one-out sensitivity analysis for pooled revision surgery rate; -**f.** Leave-one-out sensitivity analysis for pooled dermabrasion rate.

Funnel plots for each outcome are shown in Figure S2. Visual inspection and Egger’s linear regression test did not show evidence of small-study effects for the overall complication rate (t = 0.24, df = 36, *p* = 0.8149) and dermabrasion (t = –0.70, df = 23, *p* = 0.4905). Conversely, significant publication bias was observed for flap/graft necrosis (t = 2.83, df = 36, *p* = 0.0075), wound infection/dehiscence e(t = 3.02, df = 36, *p* = 0.0047), contour irregularities/contraction (t = 2.69, df = 36, *p* = 0.0107), and revision surgery (t = 4.17, df = 23, *p* = 0.0004).

**Figure S2** -**a.** Funnel plot for pooled complication rate; **-b.** Funnel plot for pooled flap/graft necrosis rate; **-c.** Funnel plot for pooled wound infection/dehiscence rate; **-d.** Funnel plot for pooled contour irregularities rate; **-e.** Funnel plot for pooled revision surgery rate; **-f.** Funnel plot for pooled dermabrasion rate.

## 4. Discussion

To the best of our knowledge, this is the first systematic review and meta-analysis to provide a comprehensive assessment of aesthetic outcomes, complication rates and revision surgery across the full spectrum of reconstructive techniques for distal nasal reconstruction.

The distal nose represents a critical functional and aesthetic subunit. Partial defects or nasal absence may result in significant structural and aesthetic deformities [1,2]. A wide range of reconstructive techniques has been described, from primary closure and skin grafts to local, regional and free flaps. Locoregional flaps continue to play a pivotal role in the reconstruction of soft-tissue defects of the head and neck, providing reliable vascularity and optimal skin matching [91]. Patient-related factors—including age, comorbidities and defect size, shape and orientation—are crucial in guiding the choice of reconstruction [92]. While small or superficial defects can often be managed with skin grafts or local flaps [6], larger or multi-subunit defects typically require more complex regional or free flap procedures [6,7]. Each technique presents specific advantages and limitations. Local flaps, such as bilobed flaps, provide a reliable single-stage coverage but may result in contour irregularities [67,78]. Nasolabial flaps offer excellent outcomes, particularly in elderly patients, although secondary refinements may be required [72,87]. Forehead flaps remain the gold standard for large or distal defects, often performed in two or three stages [46,47,48]. Among local flaps, bilobed and trilobed transposition flaps are technically demanding and geometrically complex, challenging for less experienced surgeons [39,44]. In contrast, rotation flaps may be easier to perform and less prone to pincushioning compared with transposition flaps [58,90]. Free flaps are generally reserved for extensive, full-thickness defects but may be of limited application in relation to elderly or comorbid patients due to the complexity of multistage procedures [56]. Skin grafts and composite grafts provide an alternative for selected defects, achieving satisfactory results [15,62].

In our meta-analysis, the overall complication rate after distal nasal reconstruction was 11%, with the highest rates observed in the regional flap (26%) and mixed/graft groups (17%), while local flaps demonstrated the lowest rate (~4%) and no cases of flap necrosis or wound infection/dehiscence, highlighting their safety for small to moderate defects. The free flap group showed a slightly higher complication rate (5%), but the small sample size (n = 12) limits the reliability of this estimate. Flap/graft necrosis, wound infection/dehiscence, and contour irregularities/contraction were generally rare, with necrosis absent in local and graft flaps, and contour irregularities most frequent in regional flaps (11%). Flap/graft necrosis, wound infection/dehiscence, and contour irregularities/contraction were generally rare, with necrosis absent in local and graft flaps, and contour irregularities most frequent in regional flaps (11%).

Notably, none of the studies specified whether complications such as wound infection/dehiscence or contour irregularities/contraction occurred at the donor or recipient site. This lack of granularity in reporting may affect the interpretation and clinical significance of the pooled complication rates and should be considered when evaluating the overall safety of the reconstructive techniques.

Revision surgery occurred in 7% overall, highest in mixed (11%) and regional (9%) flaps, and very rare in local and graft flaps. Dermabrasion was uncommon, with 0% pooled in the regional and mixed flap groups and 3% in the local and graft groups. Other revision treatments, such as intralesional corticosteroids or laser therapy, were rarely reported, preventing pooled analysis.

Overall, these results emphasise that local flaps are safe and reliable for small nasal defects, whereas regional and free flaps, although associated with higher complication and revision rates, remain necessary for larger or more complex defects.

The aesthetic outcomes were generally favourable across flap types. Local flaps typically achieved satisfactory to excellent results. Nasolabial flaps were generally associated with high patient satisfaction, although some variability was observed depending on the specific study. Paramedian forehead flaps demonstrated excellent aesthetic outcomes in most cases. Lu X et al. [52], compared different types of flaps (forehead, nasolabial and medial upper arm tube) and reported excellent results forehead flaps, satisfactory outcomes for nasolabial flaps, with an excellent shape, colour, texture and survival, but some bloating in relation to medial upper arm tube flaps. Free flaps, including ear helix and cartilage batten grafts, achieved a good to excellent colour and texture match, with a high overall patient satisfaction.

Regarding functional outcomes, only two studies reported nasal valve collapse or obstruction. Knackstedt T et al. [67] documented this complication following bilobed or trilobed flaps, while Pelster MW et al. [65], observed temporary nasal valve compromise after skin graft reconstruction. The limited reporting precluded any quantitative analysis, highlighting the paucity of data on functional outcomes in relation to distal nasal reconstruction.

The follow-up duration varied widely, ranging from 1 month to 17 years, with most studies reporting a duration of 6–32 months. This variability limits any direct comparison of long-term outcomes and highlights the need for a standardised, extended follow-up. The characteristics of the defect—size and thickness—directly influenced flap selection. Small defects (<2 cm) were typically managed with local flaps, providing a reliable single-stage coverage with minimal donor morbidity. Medium-sized defects, often partial thickness or single-subunit, were frequently reconstructed with nasolabial flaps or skin grafts, balancing aesthetics and vascular reliability. Large or full-thickness defects, particularly those involving multiple subunits, required regional flaps or free tissue transfer to restore structural integrity and contouring. Thickness also guided the choice of flap: superficial defects could be managed with thin local flaps or grafts, while full-thickness defects necessitated flaps with sufficient bulk, often staged, to maintain nasal projection and airway function.

In line with the GRADE framework, the certainty of evidence for the primary outcomes—overall complication rate, flap/graft necrosis, and revision surgery—was assessed and is summarised in Supplementary Table S2, providing a structured evaluation of the confidence in our pooled estimates.

Several limitations should be considered when interpreting the findings of this study. First, the studies included were heterogeneous in terms of design, sample size, follow-up duration and reporting methods, which has contributed to a substantial variability in some outcomes. Secondly, many studies did not specify whether complications, such as wound infection, dehiscence or contour irregularities, referred to the donor or recipient site, limiting the precision of complication assessment. Thirdly, the aesthetic outcomes were inconsistently reported, often using different scales or qualitative descriptions, which prevented a quantitative synthesis of these results. Furthermore, rare complications—including alar notching, nasal valve collapse and the need for dermabrasion—were reported in only a few studies, making it impossible to calculate pooled estimates. Finally, differences in flap selection criteria across the studies, influenced by defect size, location, patient comorbidities and surgeon preference, may have introduced selection bias. Taken together, these limitations highlight the need for standardised outcome reporting and further prospective, well-designed studies in this field.

The aim of this systematic review and meta-analysis was to provide an evidence-based overview of functional and aesthetic outcomes following distal nasal reconstruction across different flap techniques, offering a comparative framework rather than favouring a single approach. The findings were consistent with expected clinical trends: free flaps, although associated with a low complication rate (5%), required revision in all reported cases (100%), likely reflecting the complexity of these reconstructions and the small sample size (n = 12). Local flaps confirmed their well-established safety and reliability, showing the lowest complication and revision rates and representing the preferred option for small-to-moderate defects. Regional flaps offered an intermediate solution, balancing tissue coverage with a moderate risk of complications. Overall, while all reconstructive strategies can achieve satisfactory aesthetic results, optimal outcomes depend on careful flap selection tailored to defect size, location, and patient-specific factors. The heterogeneity of reporting and the limited number of events for some outcomes underscore the need for prospective studies with standardised evaluation of both aesthetic and functional parameters.

## Figures and Tables

**Table 1:** Qualitative assessment of included studies. RSP = retrospective; BCC = basal cell carcinoma; SCC = squamous cell carcinoma; AVM = arteriovenous malformation; * = median.

Author, Year	Design	Country	Department	Surgeon	Years	No Patients (Male)	Mean Age (Range)	Aetiology	Type of Cutaneous Reconstruction	Defect Location	Defect Depth	Mean Defect Size (Range)	Operation Stages	Mean Follow Up Time (Range)	Recurrence Rate	**Outcomes**
**Rickstrew J, 2024 [58]**	RSP	Texas, USA	Dermatology	Two	2018–2022	66 (46)	68.2	53: BCC; 11: SCC; 1: melanoma in situ; 1: desmoplastic trichoepithelioma	Nasal tip rotation flap	29: tip: 29: ala; 5: soft triangle; 3: tip	N/A	1.2 cm (0.7–1.9)	1	N/A	N/A	Complications and revisions needed
**Asaka A, 2023 [59]**	RSP	Japan	Plastic and Reconstructive Surgery	N/A	2013–2019	15 (6)	68.9 (51–86)	10: BCC; 2: keratocanthosis; 1: nevus cell; 1: trichoblastoma; 1: angioleiomyoma	Modified trilobed flap	12: tip; 2: tip and left triangle; 1: tip and columella	N/A	1.5 × 1.4 cm (1.1 × 1.1–2 × 2)	1	15.6 mo	0	Complications, patients’ satisfaction, tip’s symmetry
**He A, 2022 [60]**	RSP	China	Plastic and Reconstructive Surgery	N/A	2017–2019	13 (9)	50 (37–68)	Trauma or tumour	Postauricular skin-fat-fascia composite graft	N/A	Partial thickness	2.45 cm (2–2.9)	1	(3–14) mo	N/A	Complications and aesthetic results
**Ding F, 2021 [61]**	RSP	China	Plastic and Reconstructive Surgery	N/A	2016–2018	6 (0)	(4–24)	Benign skin tumours	Extended forehead flap	2: tip; 1: columella; 1: ala; 2: multiunit	N/A	1.5 × 2–11 × 10 cm (with the right palpebral and the cheek involved)	2 (3 weeks)	9–17 mo	N/A	Surgical and laser complications
**Kim DJ, 2021 [62]**	RSP	California, USA	Dermatology	Single and senior	2011–2018	129	70 * (33–97)	N/A	Free cartilage batten grafting (FCBG) flap	Ala	112: superficial; 14: extended to cartilage; 2: full thickness	0.8 cm^2^ * (0.15–11.1)	1	9 w	0	Complications, alar rim retraction, healed skin surface contour and aesthetic results
**Gostian AO, 2020 [63]**	RSP	Germany	Otolaryngology	N/A	2016–2018	16 (9)	68 (40–91)	9: BCC; 6: SCC; 1: trauma	Skin graft + extended forehead flap	Ala (14: multiunit)	Full thickness	N/A	3 (2nd: 5.4 w; 3rd: 10.5 w)	18.4 mo (3–35)	N/A	Complications
**Yildiz K, 2020 [64]**	N/A	Turkey	Plastic and Reconstructive Surgery	N//A	2011–2016	13 (8)	61 (42–85)	11: BCC; 2: infection	Lateral nasal artery perforator flap	Ala (6: multiunit)	Full thickness	2.8 cm^2^ (1.8–3.4)	1	22 mo (9–48)	N/A	Complications and revisions needed
**Pelster MW, 2019 [65]**	RSP	Texas, USA	Dermatology	Single	2013–2017	31 (11)	75.6	Skin tumour	Skin graft	Tip	N/A	1.8 ± 03 cm	1	15 w	N/A	Complications and aesthetic results
**Howe NM, 2019 [66]**	RSP	Philadelphia, USA	Dermatology	Single and senior	2009–2017	48 (23)	67 * (41–92)	42: BCC; 6: SCC	Crescentic island pedicle rotation flap	18: lower nasal sidewall; 16: tip; 14: ala	N/A	1.29 cm^2^ (0.25–3.8)	1	6 mo (1–23)	N/A	Complications and revisions needed
**Knackstedt T, 2018 [67]**	RSP	Ohio, USA	Dermatology	N/A	2009–2016	185 (89) 111(56) vs. 74 (33)	71.0 vs. 69.2	162: BCC; 21: BCC; 2: other	Bilobed vs. trilobed flap	28: inferior nasal dorsum; 19: inferior nasal sidewall; 54: supratip; 41: tip/infratip; 43: ala	N/A	1.36 cm^2^ vs. 1.32 cm^2^	1	63.5 d vs. 69.6 d	N/A	Flap design, complications and revisions needed
**Mohos G, 2018 [68]**	N/A	Hungary	Dermatology	N/A	N/A	10 (6)	77 (66–84)	BCC	Nasolabial flap	Ala	N/A	1.8 × 1.8–2.6 × 2.9 cm	1	7.5 mo (5–15)	N/A	Complication, aesthetic results and patients’ satisfaction
**Wang CY, 2018 [69]**	RSP	Missouri, USA	Dermatology	N/A	2013–2014	26	N/A	N/A	Trilobed flap	N/A	N/A	1.46 × 1.12 cm	1	90 d	N/A	Complications, alar symmetry and aesthetic results
**Funayama E, 2017 [70]**	RSP	Japan	Plastic and Reconstructive Surgery	N/A	1996–2013	6 (6)	65.3 (53–77)	4: BCC; 1: SCC; 1: recurrent pleomorphic adenoma	Paramedian forehead flap	Ala	Full thickness	N/A	2 (2–3 w)	37 mo	N/A	Complications and aesthetic results
**Redondo P, 2017 [5]**	RSP	Spain	Dermatology	Single	2004–2015	27 816)	62.8 (44–88)	19: BCC; 6: SCC; 2: lentigo maligna	Dorsal nasal flap	N/A	N/A	2.4 cm * (1.5 × 2.1–3.2 × 3.7)	1	12 mo–11 y	0	Complications and patients’ satisfaction
**Blázquez-Sánchez N, 2016 [71]**	RSP	Spain	Dermatology	N/A	2004–2011	41 (29)	67 (41–85)	35: BCC; 4: SCC; 1: lentigo maligna; 1: trigeminal trophic ulcer	Paramedian forehead flap	21: tip; 10: ala; 8: lower dorsum, lateral wall of the nose	32: superficial; 9: full thickness	2.16 cm (1.5–4)	2	70.9 mo (±23.3)	N/A	Complications, secondary procedures, and cosmetic results
**Ghassemi A, 2016 [72]**	N/A	Germany	Maxillofacial surgery	N/A	Since 2010	21 (12)	59.8 (43–84)	BCC	Reverse subcutaneous pedicled nasolabial flap + paramedian forehead (for coverage) flap	Ala	Full thickness	N/A	3 (2nd: 2 w; 3rd: 3–5 w)	1 y	5	Complications, aesthetic and function results
**Lu X, 2016 [52]**	RSP	China	Plastic and Reconstructive Surgery	N/A	2010–2015	34 (16)	18 (5–36)	Haemangiomas	28: forehead; 5: nasolabial; 1: medial upper arm tube flap	2: lower dorsum; 5: ala; 2: tip; 25: two or more subunits	N/A	N/A	All 1 except the medial upper arm tube flap: 2 (2 w)	12–36 mo	N/A	Complications, aesthetic and function results
**Ong S, 2016 [73]**	N/A	New Zealand	Dermatology	N/A	Past 3 years	27	64.4	Tumour	Quadrilobed flap	18: tip; 5: ala; 2: lower nasal sidewall; 2: infratip	N/A	(0.7 × 0.5–3.0 × 1.0) cm	1	N/A	N/A	Complications and revisions needed
**Scheufler O, 2016 [74]**	RSP	Switzerland	Plastic and Reconstructive Surgery	Single	2009–2015	29 (13)	73 (48–95)	N/A	Frontonasal flap (standard vs. extent)	Tip	N/A	2 cm (1.6–2.4)	1	8.4 mo	N/A	Complications and aesthetic results
**Moreno-Artero E, 2015 [75]**	N/A	Spain	Dermatology	N/A	2011–2015	15 (10)	57 (31–84)	12: BCC; 3: SCC	Spiral flap	Ala	N/A	1 cm (0.8–1.3)	1	N/A		Complications, aesthetic and functional results
**Takeda A, 2014 [76]**	RSP	Japan	Plastic and Reconstructive Surgery	N/A	N/A	5 (3)	78 (72–82)	4: BCC, 1: SCC	Nasolabial flap	Ala	Full thickness	1.9 × 1.7 cm (1.6 × 1.6–2.3 × 1.9)	1	32 mo (17–36)	0	Complications, aesthetic results revisions needed, patients’ satisfaction
**Bashir MM, 2013 [77]**	N/A	Pakistan	Plastic and Reconstructive Surgery	N/A	2007–2011	18 (6)	35 (12–24)	Trauma	Modified nasal turn-in flap + forehead flap (for coverage)	Columella, tip, soft triangles, and alae and extending partially to the dorsum and paired side wall	Full thickness	N/A	2 (3 weeks)	N/A	N/A	Airway patency and alar rim contour
**Constantine FC, 2013 [53]**	RSP	Texas, USA	Plastic and Reconstructive Surgery	Single and senior	1995–2010	14 (3)	61 (46–78)	N/A	9: nasolabial flap; 3: paramedian forehead flap; 2: skin graft	Soft triangle	2: only external skin; 9: external skin + soft tissue; 3: full thickness	N/A	N/A	N/A	N/A	Complications and revisions needed
**Hafiji J, 2012 [78]**	N/A	UK, New Zealand	Dermatology	N/A	Since 2011	45 (25)	70 (41–88)	N/A	Advancement and inferior rotation of the nasal sidewall (AIRNS) flap	The overwhelming majority of cases involved the nasal tip or the lower nasal dorsum	N/A	1.2 × 1.2 cm	1	N/A	N/A	Complications and aesthetic results
**Ibrahimi OA, 2012 [79]**	RSP	Connecticut, USA	Dermatology	N/A	N/A	16	N/A	N/A	Free cartilage batten grafting	Ala	N/A	N/A	1	N/A	N/A	Complications, aesthetic and functional results
**Mahlberg MJ, 2011 [80]**	RSP	South Carolina, USA	Dermatology	N/A	2006–2011	63 (30)	65 * (27–96)	57: BCC; 4: SCC; 1: Desmoplastic trichoepithelioma; 1: hidradenoma	Spiral flap	Ala	Partial thickness	1.0 cm (0.5–1.5)	1	Minimum 3 mo	N/A	Complications and revisions needed
**Albertini JG, 2010 [81]**	RSP	Utah, USA	Dermatology	N/A	2005–2008	31 (19)	N/A	Tumour	Trilobed flap	20: tip; 10: ala; 1: dorsum	N/A	1.6 cm	1	7 mo	N/A	Complications, alar symmetry and aesthetic results
**Tan E, 2010 [82]**	RSP	New Zealand	Dermatology	N/A	2004–2008	65 (19)	60.5 (39–86)	N/A	Nasal sidewall rotation flap	70: tip; 3: lower sidewall; 1: lower dorsum; 1: ala	N/A	1.1 × 1.0 cm (0.4–2.0)	1	Minimum 1 y	N/A	Complications and revisions needed
**Xue CY, 2009 [83]**	RSP	China	Plastic and Reconstructive Surgery	N/A	2003–2007	11 (5)	57 (42–76)	7: BCC; 2: SCC; 2: nevus cell	Bilobed flap	4: tip; 4: ala; 3: soft triangle	N/A	1.38 × 1.38 cm (1.2 × 1.2–1.6 × 1.6)	1	6 mo (1–24)	0	Complications and aesthetic results
**D’Arpa S, 2008 [84]**	RSP	Italy	Plastic and Reconstructive Surgery	N/A	2004–2007	8	73 (42–89)	7: BCC; 1: SCC	Nasolabial perforator flap	Ala	Partial thickness	N/A	1	16 mo (3–31)	N/A	Flap design, complications and aesthetic results
**Willey A, 2008 [85]**	N/A	Georgia, USA	Plastic and Reconstructive Surgery	N/A	2006–2007	61 (31)	69 (32–72)	47: BCC; 9: SCC; 3: melanoma; 1: Merckel cell carcinoma	Single-sling myocutaneous island pedicle flap	41: tip; 18: ala; 3: lower sidewall	N/A	0.6–2.8 cm	1	N/A	N/A	Complications, aesthetic and functional results
**Ozek C, 2007 [86]**	N/A	Turkey	Plastic and Reconstructive Surgery	N/A	1997–2005	6 (6)	52 (41–62)	4: BCC; 2: SCC	Ear helix free flap	2: ala + columella; 2: ala; 1: tip + ala; 1: columella	Full thickness	3.2 × 2.7 cm (2.9 × 2.5–3.6 × 3.0)	1	N/A	N/A	Complications, aesthetic results and patients’ satisfaction
**Silistreli OK, 2005 [87]**	RSP	Turkey	Plastic and Reconstructive Surgery	N/A	2000–2002	10 (5)	53.8 (15–78)	5: BCC; 1: metatypical cancer; 3: trauma; 1: congenital nevus	Nasolabial flap	5: ala; 3: columella; 1: tip	Full thickness	1.8 × 1.3 cm (1.1 × 1.0–2.5 × 1.5)	2 (2 w)	25 mo (18–31)	N/A	Complications and revisions needed
**Ullmann Y, 2005 [88]**	N/A	Israel	Plastic and Reconstructive Surgery	N/A	2002–2003	17 (6)	(46–84)	BCC/SCC	Paramedian forehead flap	N/A	3: full thickness	(1.8–3.6 cm)	2 (2–3 w)	N/A	N/A	Complications, aesthetic results and revisions needed
**Lambert RW, 2004 [89]**	N/A	Pennsylvania, USA	Dermatology	N/A	N/A	30	N/A	N/A	Dorsal nasal advancement flap	Tip	N/A	0.5 × 2.0 cm	1	N/A	N/A	Complications and aesthetic results
**Lindsey WH, 2001 [90]**	RSP	Virginia, USA	Otolaryngology	Single	Dating back 41⁄2 years	105	N/A	Tumour	Nasolabial flap	Ala	17: full thickness	N/A	1	Minimum 6 mo	N/A	Complications and revisions needed
**Goleman R, 1998 [54]**	N/A	Brazil	Plastic and Reconstructive Surgery	N/A	N/A	21 (9)	62.71 (25–83)	19: BCC; 1: SCC; 1: actinic keratosis	Bilobed island flap	Ala	19: partial thickness; 2: full thickness	1.6 × 1.7 cm (1.1 × 1.2–2.1 × 2)	1	N/A	N/A	Flap design and complications
**Blandini D, 1996 [55]**	N/A	Italy	Plastic and Reconstructive Surgery	N/A	17 y	53 (21)	65.7 (49–97)	35: BCC; 13: SCC; 5: pigmented lesion	Axial sliding dorsal nasal flap	Ala	N/A	1 × 1 cm- entire lobular	1	47.3 mo (3 mo–17 y)	N/A	Complications
**Pribaz JJ, 1993 [56]**	RSP	Massachusetts, USA	Plastic and Reconstructive Surgery	N/A	1985–1992	6 (2)	43 (7–76)	2: BCC; 2: AVM; 1: sarcoma; 1: huma bite	Auricular free flap	3: ala; 2: columella; 1: two or more subunits	N/A	N/A	N/A	N/A	N/A	Complications and revisions needed
**Wee SS, 1990 [57]**	N/A	Missouri, USA	Dermatology	Senior and single	3 y	19 (8)	(30–76)	18: BCC; 1: lentigo maligna	Nasalis myocutaneous island flap	Ala	N/A	1 × 0.9–4 × 3.5 cm	1	1 mo–2.5 y	0	Complications and aesthetic results

**Table 2:** Complications reported in each included study.

Author, Year	Type of Cutaneous Reconstruction	No Patients	Complications							
			Tot	*Flap/Graft Necrosis*	*Wound Infection/Dehiscence*	*Alar Notching*	*Contour Irregularities/Contraction*	*Nasal Valve Collapse/Obstruction*	*Pincushion/Trapdoor*	*Others*
**Rickstrew J, 2024 [58]**	Nasal tip rotation flap	66	**5**	0	0	2	3	0	0	0
**Asaka A, 2023 [59]**	Modified trilobed flap	15	**0**							
**He A, 2022 [60]**	Postauricular skin-fat-fascia composite graft	13	**0**							
**Ding F, 2021 [61]**	Extended forehead flap	6	**0**							
**Kim DJ, 2021 [62]**	Free cartilage batten grafting (FCBG) flap	129	**1**	0	0	0	1	0	0	0
**Gostian AO, 2020 [63]**	Skin graft + extended forehead flap	16	**4**	1 (partial)	1	0	0	0	0	1: pain; 1: perforation of nasal septum
**Yildiz K, 2020 [64]**	Lateral nasal artery perforator flap	13	**1**	0	0	0	1	0	0	0
**Pelster MW, 2019 [65]**	Skin graft	31	**5**	0	3	0	0	1 (temporary)	0	1: bleeding
**Howe NM, 2019 [66]**	Crescentic island pedicle rotation flap	48	**6**	0	1	1	4	0	0	0
**Knackstedt T, 2018 [67]**	Bilobed vs. trilobed flap	185	**57**	0	0	0	0	3	44	10: erythema;
**Mohos G, 2018 [68]**	Nasolabial flap	10	**5**	0	0	0	0	0	0	3: sweeling; 2: erythema
**Wang CY, 2018 [69]**	Trilobed flap	26	**3**	0	0	0	2	0	1	0
**Funayama E, 2017 [70]**	Paramedian forehead flap	6	**5**	0	0	0	5	0	0	0
**Redondo P, 2017 [5]**	Dorsal nasal flap	27	**0**							
**Blázquez-Sánchez N, 2016 [71]**	Paramedian forehead flap	41	**28**	5	1	0	15	0	0	8: transposition of forehead hair with the flap
**Ghassemi A, 2016 [72]**	Reverse subcutaneous pedicled nasolabial flap + paramedian forehead flap	21	**1**	0	1	0	0	0	0	0
**Lu X, 2016 [52]**	28: forehead; 5: nasolabial; 1: medial upper arm tube flap	34	**1**	0	0	0	0	0	0	1: swelling
**Ong S, 2016 [73]**	Quadrilobed flap	27	**0**							
**Scheufler O, 2016 [74]**	Frontonasal flap (standard vs. extent)	29	**8**	0	5	0	2	0	0	1: haematoma
**Moreno-Artero E, 2015 [75]**	Spiral flap	15	**1**	0	0	0	1	0	0	0
**Takeda A, 2014 [76]**	Nasolabial flap	5	**1**	0	0	0	1	0	0	0
**Bashir MM, 2013 [77]**	Modified nasal turn-in flap + forehead flap (for coverage)	18	**5**	5	0	0	0	0	0	0
**Constantine FC, 2013 [53]**	9: nasolabial flap; 3: paramedian forehead flap; 2: skin graft	14	**N/A**							
**Hafiji J, 2012 [78]**	Advancement and inferior rotation of the nasal sidewall (AIRNS) flap	45	**1**	0	1	0	0	0	0	0
**Ibrahimi OA, 2012 [79]**	Free cartilage batten grafting	16	**13**	0	3	2	6	0	0	2: pain
**Mahlberg MJ, 2011 [80]**	Spiral flap	63	**0**							
**Albertini JG, 2010 [81]**	Trilobed flap	31 (19)	**0**							
**Tan E, 2010 [82]**	Nasal sidewall rotation flap	65 (19)	**11**	1	0	0	1	0	0	9: swelling
**Xue CY, 2009 [83]**	Bilobed flap	11 (5)	**0**							
**D’Arpa S, 2008 [84]**	Nasolabial perforator flap	8	**2**	1	0	0	0	0	2	0
**Willey A, 2008 [85]**	Single-sling myocutaneous island pedicle flap	61 (31)	**12**	0	2	1	8	0	0	1: haemorrhage
**Ozek C, 2007 [86]**	Ear helix free flap	6 (6)	**0**							
**Silistreli OK, 2005 [87]**	Nasolabial flap	10 (5)	**3**	1 (partial)	0	0	2	0	0	0
**Ullmann Y, 2005 [88]**	Paramedian forehead flap	17 (6)	**3**	1 (partial)	2	0	0	0	0	0
**Lambert RW, 2004 [89]**	Dorsal nasal advancement flap	30	**0**							
**Lindsey WH, 2001 [90]**	Nasolabial flap	105	**16**	7 (partial)	4	0	0	0	0	4: haematoma
**Golcman R, 1998 [54]**	Bilobed island flap	21 (9)	**1**	0	1	0	0	0	0	0
**Blandini D, 1996 [55]**	Axial sliding dorsal nasal flap	53 (21)	**0**							
**Pribaz JJ, 1993 [56]**	Auricular free flap	6 (2)	**1**	0	0	0	1	0	0	0
**Wee SS, 1990 [57]**	Nasalis myocutaneous island flap	19	**0**							

**Table 3:** Overview of revision surgeries in each included study.

Author, Year	Type of Cutaneous Reconstruction	No Patients	Complications	Revision Surgery	Dermabrasion	Intralesional Corticosteroid Injection	Laser	Comment
**Rickstrew J, 2024 [58]**	Nasal tip rotation flap	66	5	0	2	1	0	
**Kim DJ, 2021 [62]**	Free cartilage batten grafting (FCBG) flap	129	1	0	10	0	0	
**Gostian AO, 2020 [63]**	Skin graft + extended forehead flap	16	4	2	0	0	0	
**Yildiz K, 2020 [64]**	Lateral nasal artery perforator flap	13	1	1	0	0	0	Flap debulking
**Pelster MW, 2019 [65]**	Skin graft	31	5	4	0	0	0	
**Howe NM, 2019 [66]**	Crescentic island pedicle rotation flap	48	6	0	1	2	0	
**Knackstedt T, 2018 [67]**	Bilobed vs. trilobed flap	185	57	2	4	44	10	
**Mohos G, 2018 [68]**	Nasolabial flap	10	5	0	0	0	0	
**Wang CY, 2018 [69]**	Trilobed flap	26	3	4	1	0	0	
**Funayama E, 2017 [70]**	Paramedian forehead flap	6	5	5	0	0	0	Flap debulking
**Blázquez-Sánchez N, 2016 [71]**	Paramedian forehead flap	41	28	7	3	0	0	Flap debulking and hair removal
**Ghassemi A, 2016 [72]**	Reverse subcutaneous pedicled nasolabial flap + paramedian forehead flap	21	1	3	0	0	0	
**Lu X, 2016 [52]**	28: forehead; 5: nasolabial; 1: medial upper arm tube flap	34	1	1	0	0	0	
**Scheufler O, 2016 [74]**	Frontonasal flap (standard vs. extent)	29	8	4	0	0	0	2: flap debulking; 1: haematoma evacuation; 1: scar revision
**Moreno-Artero E, 2015 [75]**	Spiral flap	15	1	0	0	0	0	
**Takeda A, 2014 [76]**	Nasolabial flap	5	1	1 (minor)	0	0	0	
**Bashir MM, 2013 [77]**	Modified nasal turn-in flap + forehead flap (for coverage)	18	5	1	0	0	0	
**Constantine FC, 2013 [53]**	9: nasolabial flap; 3: paramedian forehead flap; 2: skin graft	14	N/A	3	5	0	0	3 required revisions with subsequent resurfacing, 2 required resurfacing alone.
**Hafiji J, 2012 [78]**	Advancement and inferior rotation of the nasal sidewall (AIRNS) flap	45	1	0	0	0	0	
**Ibrahimi OA, 2012 [79]**	Free cartilage batten grafting	16	13	N/A	N/A	N/A	N/A	
**Tan E, 2010 [82]**	Nasal sidewall rotation flap	65 (19)	11	0	3	9	0	
**D’Arpa S, 2008 [84]**	Nasolabial perforator flap	8	2	0	0	0	0	
**Willey A, 2008 [85]**	Single-sling myocutaneous island pedicle flap	61 (31)	12	0	8	0	0	
**Silistreli OK, 2005 [87]**	Nasolabial flap	10 (5)	3	0	0	0	0	
**Ullmann Y, 2005 [88]**	Paramedian forehead flap	17 (6)	3	10	0	0	0	3 patients require 2 revision surgeries each, while 7 patients required 1 revision surgery each
**Lindsey WH, 2001 [90]**	Nasolabial flap	105	16	1	0	15	0	
**Goleman R, 1998 [54]**	Bilobed island flap	21 (9)	1	0	0	0	0	
**Pribaz JJ, 1993 [56]**	Auricular free flap	6 (2)	1	6 (minor)	0	0	0	

**Table 4:** Qualitative assessment of aesthetic outcomes reported in each included study.

Author, Year	Type of Cutaneous Reconstruction	Aesthetic Results
**Rickstrew J, 2024 [58]**	Nasal tip rotation flap	N/A
**Asaka A, 2023 [59]**	Modified trilobed flap	Good colour and texture all
**He A, 2022 [60]**	Postauricular skin-fat-fascia composite graft	Favourable
**Ding F, 2021 [61]**	Extended forehead flap	Good colour and texture all
**Kim DJ, 2021 [62]**	Free cartilage batten grafting (FCBG) flap	18: poor; 40: good; 40: very good; 31: excellent
**Gostian AO, 2020 [63]**	Skin graft + extended forehead flap	N/A
**Yildiz K, 2020 [64]**	Lateral nasal artery perforator flap	N/A
**Mohos G, 2018 [68]**	Nasolabial flap	All satisfied
**Pelster MW, 2019 [65]**	Skin graft	All satisfied
**Howe NM, 2019 [66]**	Crescentic island pedicle rotation flap	N/A
**Knackstedt T, 2018 [67]**	Bilobed vs. trilobed flap	N/A
**Wang CY, 2018 [69]**	Trilobed flap	Excellent-very good
**Redondo P, 2017 [5]**	Dorsal nasal flap	Good
**Funayama E, 2017 [70]**	Paramedian forehead flap	4: excellent, 1: good, 1: fair
**Lu X, 2016 [52]**	28: forehead; 5: nasolabial; 1: medial upper arm tube flap	Forehead: excellent; Nasolabial: satisfying, shape, colour, texture was excellent; Upper arm tube flap: survived but bloated
**Blázquez-Sánchez N, 2016 [71]**	Paramedian forehead flap	4: unacceptable; 19: acceptable; 18: excellent
**Ghassemi A, 2016 68]**	Reverse subcutaneous pedicled nasolabial flap + paramedian forehead (for coverage) flap	19: satisfied; 2: unsatisfied
**Ong S, 2016 [73]**	Quadrilobed flap	N/A
**Scheufler O, 2016 [74]**	Frontonasal flap (standard vs. extent)	28: good, excellent
**Moreno-Artero E, 2015 [75]**	Spiral flap	Adequate
**Takeda A, 2014 [76]**	Nasolabial flap	Acceptable
**Bashir MM, 2013 [77]**	Modified nasal turn-in flap + forehead flap	3: satisfactory; 9: just satisfactory; 6: unsatisfactory
**Constantine FC, 2013 [53]**	9: nasolabial flap; 3: paramedian forehead flap; 2: skin graft	N/A
**Hafiji J, 2012 [78]**	Advancement and inferior rotation of the nasal sidewall (AIRNS) flap	Good or excellent
**Mahlberg MJ, 2011 [80]**	Spiral flap	N/A
**Ibrahimi OA, 2012 [79]**	Free cartilage batten grafting	7: excellent, 6: very good, 3: good
**Albertini JG, 2010 [81]**	Trilobed flap	0.84 (range 0–3)
**Tan E, 2010 [82]**	Nasal sidewall rotation flap	N/A
**Xue CY, 2009 [83]**	Bilobed flap	Excellent
**D’Arpa S, 2008 [84]**	Nasolabial perforator flap	All satisfied
**Willey A, 2008 [85]**	Single-sling myocutaneous island pedicle flap	High aesthetic goals were achieved
**Ozek C, 2007 [86]**	Ear helix free flap	Good colour match
**Silistreli OK, 2005 [87]**	Nasolabial flap	N/A
**Ullmann Y, 2005 [88]**	Paramedian forehead flap	All satisfied
**Lambert RW, 2004 [89]**	Dorsal nasal advancement flap	Good outstanding
**Lindsey WH, 2001 [90]**	Nasolabial flap	N/A
**Goleman R, 1998 [54]**	Bilobed island flap	N/A
**Blandini D, 1996 [55]**	Axial sliding dorsal nasal flap	N/A
**Pribaz JJ, 1993 [56]**	Auricular free flap	N/A
**Wee SS, 1990 [57]**	Nasalis myocutaneous island flap	Excellent

## Data Availability

Not applicable.

## Supplementary Materials

The following supporting information can be downloaded at: https://www.mdpi.com/article/10.3390/jcm14227983/s1, Table S1: Complete search strategy. Table S2: GRADE assessment. Table S3: Newcastle-Ottawa Quality Assessment Scale scores of the individual studies. Figure S1: (a) Leave-one-out sensitivity analysis for pooled complication rate; (b) Leave-one-out sensitivity analysis for pooled flap/graft necrosis rate; (c) Leave-one-out sensitivity analysis for pooled wound infection/dehiscence rate; (d) Leave-one-out sensitivity analysis for pooled contour irregularities rate; (e) Leave-one-out sensitivity analysis for pooled revision surgery rate; (f) Leave-one-out sensitivity analysis for pooled dermabrasion rate. Figure S2: (a) Funnel plot for pooled complication rate; (b) Funnel plot for pooled flap/graft necrosis rate; (c) Funnel plot for pooled wound infection/dehiscence rate; (d) Funnel plot for pooled contour irregularities rate; (e) Funnel plot for pooled revision surgery rate; (f) Funnel plot for pooled dermabrasion rate. File S1: PRISMA_2020_checklist.
