# Supplementary material for: Surgical Outcomes and Complications of Distal Nasal Reconstruction: A Systematic Review and Meta-Analysis
Source: J Clin Med. 2025 Nov 11;14(22):7983. doi: 10.3390/jcm14227983 (PMC12653132; doi:10.3390/jcm14227983)
Supplement: Supplementary file 1 [file jcm-14-07983-s001.zip › Supplementary Materials.pdf]

Database	Search string (complete, unabridged; including MeSH terms, keywords, field tags, Boolean operators, and filters)
**PubMed/MEDLINE**	(**“Nose”[MeSH Terms]** OR “nasal”[All Fields]) AND (“reconstruction”[All Fields] OR “reconstructive surgical procedures”[MeSH Terms] OR “flap”[All Fields] OR “graft”[All Fields]) AND (“distal”[All Fields] OR “tip”[All Fields] OR “ala”[All Fields] OR “alar”[All Fields]) AND (“outcomes”[All Fields] OR “complications”[All Fields] OR “necrosis”[All Fields] OR “infection”[All Fields]) Filters: Humans, English, no date restrictions
**Embase (Ovid)**	('nose reconstruction'/exp OR 'nasal reconstruction':ab,ti OR 'nasal flap':ab,ti OR 'forehead flap':ab,ti OR 'nasolabial flap':ab,ti) AND ('distal nose':ab,ti OR 'nasal tip':ab,ti OR 'alar':ab,ti) AND ('complication':ab,ti OR 'outcome':ab,ti OR 'necrosis':ab,ti OR 'infection':ab,ti)
**Scopus**	(TITLE-ABS-KEY((nasal OR nose) AND (reconstruction OR flap OR graft) AND (distal OR tip OR ala OR alar) AND (outcome OR complication OR necrosis OR infection)))
**Cochrane Library**	(nasal OR nose) in Title Abstract Keyword AND (reconstruction OR flap OR graft) in Title Abstract Keyword AND (distal OR tip OR ala OR alar) in Title Abstract Keyword
**Google Scholar**	“distal nasal reconstruction” OR “nasal tip reconstruction” OR “forehead flap” OR “nasolabial flap” OR “bilobed flap” AND (outcomes OR complications) — first 200 results screened

Table S1. Complete search strategy.

Outcome	No. of Studies	No. of Patients	Effect Estimate (95% CI)	Certainty of Evidence (GRADE)	Key Reasons for Rating
Complication rate	38	1362	11% (8–14%)	Moderate ⊕⊕⊕◯	Risk of bias in included studies; some heterogeneity
Flap/graft necrosis	35	1200	3% (2–5%)	High ⊕⊕⊕⊕	Consistent results, low risk of bias
Revision surgery	36	1300	7% (5–10%)	Moderate ⊕⊕⊕◯	Some inconsistency among studies; imprecision in estimates

Table S2. GRADE assesment.

Author, Year	Selection	Comparison	Outcome
**Rickstrew J, 2024**	xxx	x	xx
**Asaka A, 2023**	xxx	xx	xx
**He A, 2022**	xxx	x	xxx
**Ding F, 2021**	xxx	x	xxx
**Kim DJ, 2021**	xxx	x	xxx
**Gostian AO, 2020**	xxx	x	xx
**Yildiz K, 2020**	xxx	x	xx
**Pelster MW, 2019**	xxx	x	xxx
**Howe NM, 2019**	xxx	x	xxx
**Knackstedt T, 2018**	xxx	xx	xx
**Mohos G, 2018**	xxx	x	xxx
**Wang CY, 2018**	xxx	x	xxx

**Funayama E, 2017**	xxx	x	xxx
**Redondo P, 2017**	xxx	x	xxx
**Blázquez-Sánchez N, 2016**	xxx	x	xxx
**Ghassemi A, 2016**	xxx	xx	xxx
**Lu X, 2016**	xx	xx	xx
**Ong S, 2016**	xxx	x	xxx
**Scheufler O, 2016**	xxx	x	xxx
**Moreno-Artero E, 2015**	xxx	x	xxx
**Takeda A, 2014**	xxx	x	xxx
**Bashir MM, 2013**	xxx	x	xxx
**Constantine FC, 2013**	xx	xx	xx
**Hafiji J, 2012**	xxx	x	xxx
**Ibrahimi OA, 2012**	xxx	x	xxx
**Mahlberg MJ, 2011**	xxx	x	xxx
**Albertini JG, 2010**	xxx	x	xxx
**Tan E, 2010**	xxx	x	xxx
**Xue CY, 2009**	xxx	x	xxx
**D'Arpa S, 2008**	xxx	x	xxx
**Willey A, 2008**	xxx	x	xxx
**Ozek C, 2007**	xxx	x	xxx
**Silistreli OK, 2005**	xxx	x	xxx
**Ullmann Y, 2005**	xxx	x	xxx
**Lambert RW, 2004**	xxx	x	xxx
**Lindsey WH, 2001**	xx	x	xx
**Golcman R, 1998**	xx	x	xx

**Blandini D, 1996**	xx	x	xx
**Pribaz JJ, 1993**	xx	x	xx
**Wee SS, 1990**	xx	x	xx

Table S3. Newcastle-Ottawa Quality Assessment Scale scores of the individual studies.

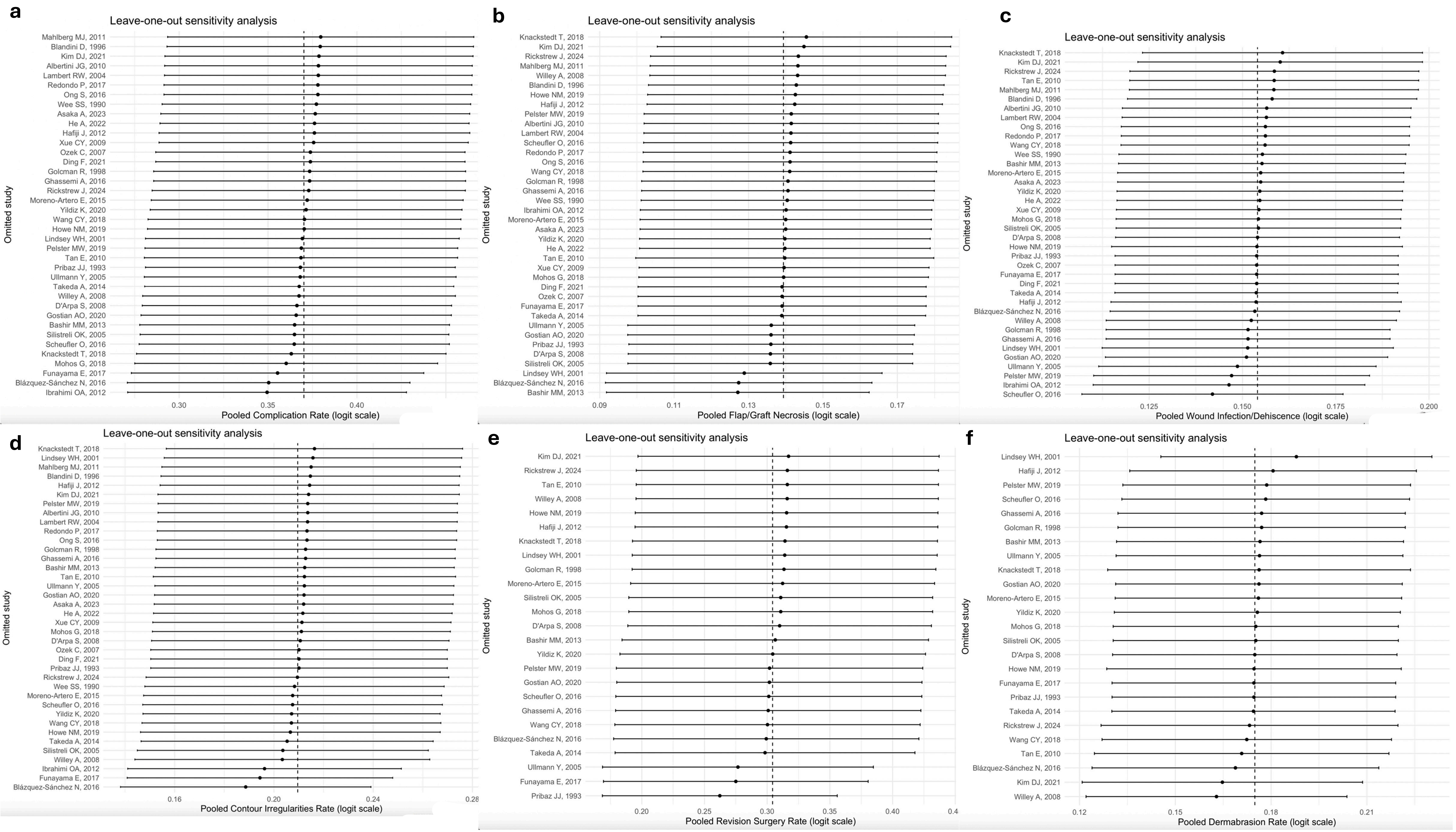

Figure S1. -a. Leave-one-out sensitivity analysis for pooled complication rate; **-b.** Leave-one-out sensitivity analysis for pooled flap/graft necrosis rate; **-c.** Leave-one-out sensitivity analysis for pooled wound infection/dehiscence rate; **-d.** Leave-one-out sensitivity analysis for pooled contour irregularities rate; **-e.** Leave-one-out sensitivity analysis for pooled revision surgery rate; **-f.** Leave-one-out sensitivity analysis for pooled dermabrasion rate.

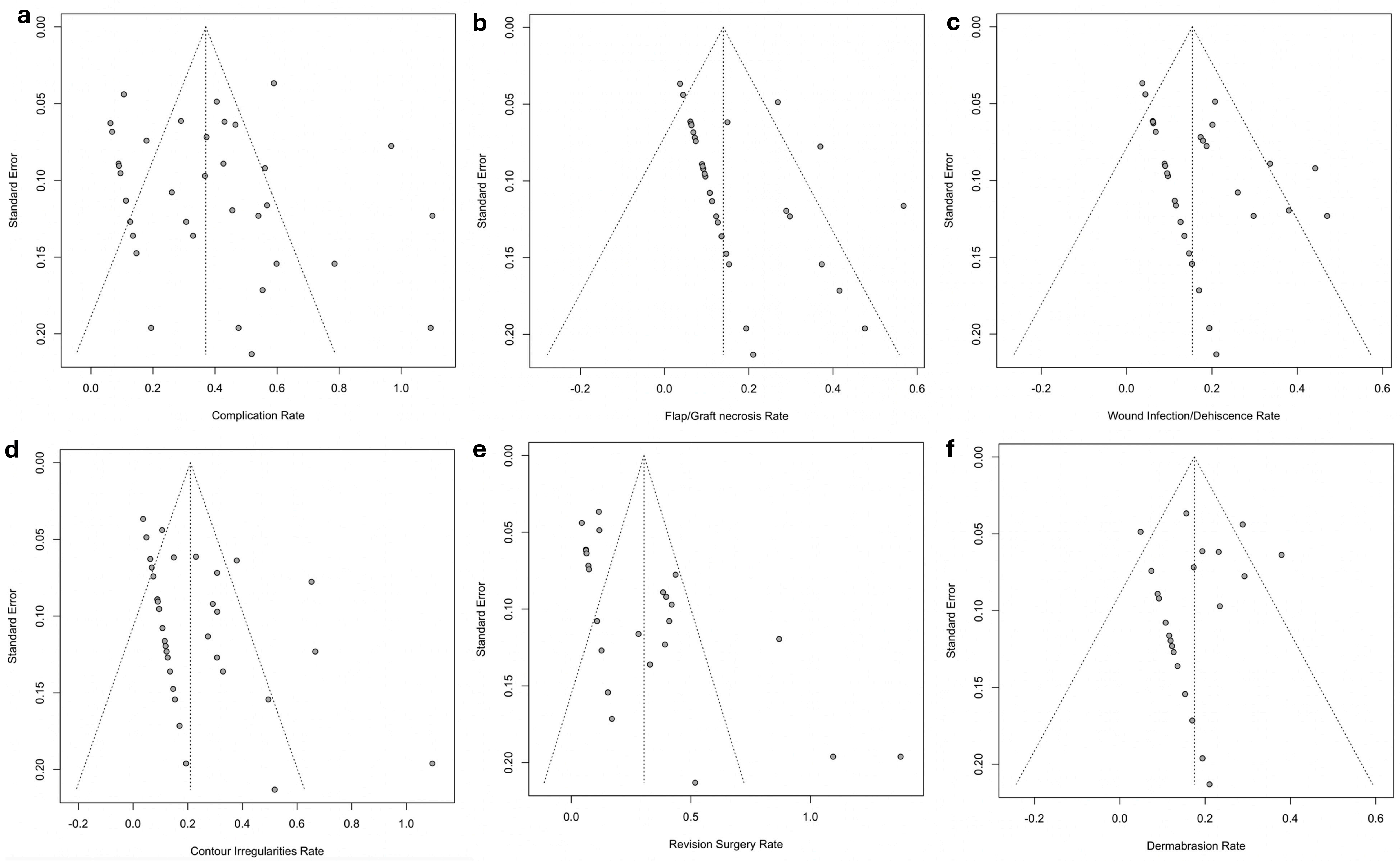

Figure S2. -a. Funnel plot for pooled complication rate; **-b.** Funnel plot for pooled flap/graft necrosis rate; **-c.** Funnel plot for pooled wound infection/dehiscence rate; **-d.** Funnel plot for for pooled contour irregularities rate; **-e.** Funnel plot for for pooled revision surgery rate; **-f.** Funnel plot for for pooled dermabrasion rate.
